# Environment-Monitoring IoT Devices Powered by a TEG Which Converts Thermal Flux between Air and Near-Surface Soil into Electrical Energy

**DOI:** 10.3390/s21238098

**Published:** 2021-12-03

**Authors:** Tereza Paterova, Michal Prauzek, Jaromir Konecny, Stepan Ozana, Petr Zmij, Martin Stankus, Dieter Weise, Alexander Pierer

**Affiliations:** 1 Department of Cybernetics and Biomedical Engineering, VSB—Technical University of Ostrava, 708 00 Ostrava, Czech Republic; tereza.paterova@vsb.cz (T.P.); jaromir.konecny@vsb.cz (J.K.); stepan.ozana@vsb.cz (S.O.); martin.stankus@vsb.cz (M.S.); 2 Brose CZ Spol. s r.o., 742 21 Koprivnice, Czech Republic; petr.zmij@brose.com; 3 Fraunhofer Institute for Machine Tools and Forming Technology IWU, 09126 Chemnitz, Germany; Dieter.Weise@iwu.fraunhofer.de (D.W.); Alexander.Pierer@iwu.fraunhofer.de (A.P.)

**Keywords:** thermoelectric generator, energy harvesting, IoT, WSN, DC/DC boost converter, LoRaWAN

## Abstract

Energy harvesting has an essential role in the development of reliable devices for environmental wireless sensor networks (EWSN) in the Internet of Things (IoT), without considering the need to replace discharged batteries. Thermoelectric energy is a renewable energy source that can be exploited in order to efficiently charge a battery. The paper presents a simulation of an environment monitoring device powered by a thermoelectric generator (TEG) that harvests energy from the temperature difference between air and soil. The simulation represents a mathematical description of an EWSN, which consists of a sensor model powered by a DC/DC boost converter via a TEG and a load, which simulates data transmission, a control algorithm and data collection. The results section provides a detailed description of the harvested energy parameters and properties and their possibilities for use. The harvested energy allows supplying the load with an average power of 129.04 μW and maximum power of 752.27 μW. The first part of the results section examines the process of temperature differences and the daily amount of harvested energy. The second part of the results section provides a comprehensive analysis of various settings for the EWSN device’s operational period and sleep consumption. The study investigates the device’s number of operational cycles, quantity of energy used, discharge time, failures and overheads.

## 1. Introduction

Interest in energy harvesting has been accelerating as the number of applications deployed long term in wireless sensor networks (WSN) and the Internet of Things (IoT) grows and the economic advantages over battery-based energy sources become more prevalent [1,2,3]. The topic of energy harvesting also relates to a subgroup of WSN called environmental WSN (EWSN) [4]. WSNs are typically used to obtain information to monitor the chemical, biological or population-related parameters of the environments under surveillance [5,6]. EWSNs are often deployed in remote areas, obstructing power supply from power grids and impeding regular battery subsystem maintenance [7]. Sources that provide energy through energy harvesting to operate wireless sensor nodes in environmental applications include solar radiation, temperature differences, flow-based systems (e.g., wind power), kinetic energy and radio frequency (RF) [8,9,10,11].

This contribution investigates thermal energy that can be converted into electrical energy by a thermoelectric generator (TEG) using spatial variations in temperature [12]. Thermoelectric transducers and generators are based on the Seebeck effect [13] and are composed of several pairs of p-type and n-type semiconductor blocks ordered in parallel and connected electrically in series [12]. The open circuit voltage of a thermoelectric element depends on the temperature difference (ΔT) between a hot and a cold surface and also on material properties called Seebeck coefficients [14,15]. The authors in [16] verified that TEG is a suitable solution for powering energy harvesting nodes. TEG can be also used to extend the battery life of devices by generating power from waste heat [17,18,19].

Thermal energy harvesting subsystems have been applied in many other outdoor applications; these are summarized in Table 1, which describes the state-of-the-art. The presented article explores TEG applications, which can directly convert temperature differences ΔT between soil and near-surface air. This TEG energy harvesting method, which exploits air and soil, has been studied in state-of-the-art publications listed in Table 2; these publications were used as references for the experiment proposed in the current study.

The presented paper provides and investigates a simulation of an environment-monitoring IoT device powered by a TEG that produces energy through the temperature difference between air and soil. The TEG power source is connected to a boost converter, which links the generated power to the system’s load, an energy storage unit (supercapacitor) and an embedded sensor (e.g., a temperature sensor to measure the temperature of an environment) that measures a certain parameter in the environment. A novelty of the current paper is presented in the use of the finite element method (FEM) to simulate the temperature difference between the lower and upper surfaces of the TEG in connection with the hardware consumption model. The obtained temperature gradient is used to drive simulation, including energy conversion and consumption model.

A major motivation of this study is the design of a rapid prototyping procedure that can evaluate advanced designs of TEG powered environmental sensors without any necessary infield deployment. The first step of the rapid prototyping FEM method provides very fast paced evaluation about the efficiency of the proposed hardware solution containing TEG energy harvesting element based on historical data from particular deployment location. Next, the energy converter can optimize hardware configuration capacitors and converter circuits. The last model of the EWSN node is powered by incoming harvested energy, and it can implement advanced energy management strategies based on machine learning in the future. The authors now present a simulation study that evaluates a rapid prototyping procedure; in the future, this approach allows the effective design of TEG-powered EWSN devices.

The paper is organized as follows: Section 2 describes related studies to this particular contribution. Section 3 provides the theoretical background to TEGs and boost converters and a description of temperature differences between air and soil. Section 4 describes an experiment with a module, e.g., sensor simulation and each part of the presented module and historical input data. Section 5 contains the results of the proposed experiments. Section 6 provides a discussion of the final results. Section 7 provides conclusions and a summary of the overall contributions of the presented paper and outlines potential challenges in future studies.

## 2. Related Work

Thermal energy is used in many outdoor applications as a reliable power source since it offers a less environmentally degrading means of obtaining power by reusing or recycling waste thermal energy. Another reason for the abundant application of TEGs is their capability of powering low-power electronics by exploiting even very small (only a few degrees Celsius) temperature gradients. Table 1 lists some applications and types of TEG in the state of the art.

Datta et al. [20] developed a device prototype for harvesting thermoelectric energy from heat on asphalt road and pavement surfaces. The energy was transferred to a TEG (TXL-287-03Z) embedded at the edge of the road surface. The results showed that the produced energy is sufficient for powering pavement health monitoring and roadway communications devices in off-grid areas [20]. The device can be also used as an alternative source for self-powered electronic road signs and markings and LED street illumination in remote areas.

The authors of [21] explored a novel approach for harvesting energy from roads and pavements, demonstrating the possibility of powering LED traffic lights and wireless sensors embedded into pavement structures from a TEG (SP1848) [21].

Rudolph et al. [22] developed a simulation that evaluated the possibility of powering a wireless sensor node using TEG technology. The authors connected a heat exchanger to a TEG to use the waste heat produced by a heavy-duty truck [22]. Yang et al. [23] proposed and developed a thermal management system that harvests and recycles the thermal waste of high-power light-emitting diodes (HP-LED) using a TEG that contains $Bi_{2}Te_{3}$ [23]. A voltage boost converter (LTC3108) was used to aid in powering a temperature sensor that monitored the surface temperature of the HP-LED.

Priya et al. [24] developed a thermal energy harvesting regulator driven by human body heat. The device contains a TEG and is suitable for powering compact, wearable biomedical IoT nodes with many features. It is ideal for sensors that can be worn on the body or low-power IoT systems that require more power for enhanced features yet have practical limitations in employing intermediate power storage between charging and regulating cycles [24].

Praveena et al. [25] used a TEG module containing $BhTe_{3}$ and $Sb_{2}Te_{3}$ to generate electrical energy from the heat dissipated by a vehicle’s engine and exhaust silencer and subsequently powered IoT sensors. A DC/DC converter was used to boost the voltage to the required level since the initial produced voltage was insufficient for powering applications.

Seyoum et al. [26] presented a design for an ambient-powered wireless bolt for use in high-end electro-mechanical systems. The bolt is equipped with a temperature sensor and a low-power RF chip powered from a TEG. The bolt includes a DC/DC converter for raising the low TEG voltage and ensuring continuous wireless monitoring of these critical fasteners.

## 3. Background

This section provides a principle explanation of the individual model components: a TEG and a boost converter. This overview also describes the general regularities of temperature differences between underground soil and air.

### 3.1. Thermoelectric Generator

**Figure 1:**
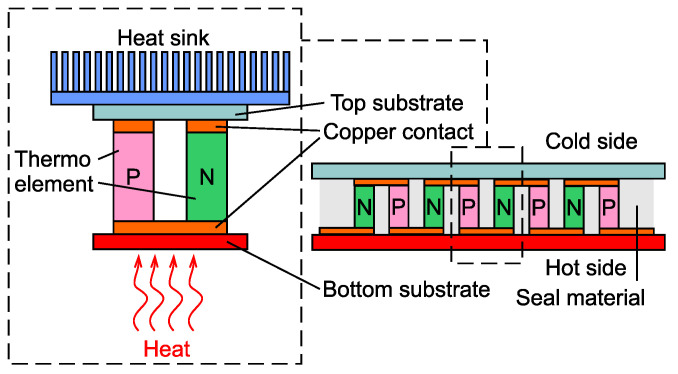
Principle scheme of a thermoelectric generator. The Seebeck effect is exploited to produce electrical energy.

A TEG is a solid-state device that is able to directly convert heat flux into electrical energy through a phenomenon called the Seebeck effect [13]. In order to generate electrical energy, the TEG consists of many n-type and p-type semiconductors sandwiched between two electrically insulating materials that possess thermoelectric properties. When one side of a TEG is heated and the other side remains cool, voltage is generated through the Seebeck effect. The TEG principle is illustrated in Figure 1.

The p-type components are doped to provide a greater number of positively charged carriers or holes, thereby providing a positive Seebeck coefficient (α). In a similar manner, n-type components are doped to provide more negatively charged carriers, thereby providing a negative α. Table 3 lists the Seebeck coefficient values for chemical elements used in TEGs.

As an electrical connection forms between the two junctions, every positively charged carrier moves to the n-junction and every negatively charged carrier moves to the p-junction. The most used element in thermoelectric generators is lead telluride (PbTe). For the flow of electrons, a suitable material with high electrical conductivity and low thermal conductivity was used. Compounds such as bismuth sulphide ($Bi_{2}S_{3}$), tin telluride (*SnTe*), bismuth telluride ($Bi_{2}Te_{3}$), indium arsenide (*InAs*) and germanium telluride (*GeTe*) are, therefore, frequently used for this purpose [30].

**Table 3:** Seebeck coefficient α values for various chemical elements (at 300 K) [31].

Element	Pb	In	Sn	Al	Au	Na	Ni	Ag
α (μV/K${}^{-1}$)	−1.05	1.68	−1	−1.66	1.94	−6.3	−19.5	1.51

Using the ΔT from the TEG and the coefficient α, the induced thermoelectric voltage $V_{\mathrm{TEG}}$ can be expressed by the following formula.

(1) $$V_{\mathrm{TEG}}=\alpha \times \Delta T.$$

A TEG has many advantages over other types of energy harvester. For example, the TEG can be used to harvest a variable amount of power according to the ΔT of the TEG. A TEG also contains no moving parts, is able to function under extreme and zero gravitational forces, exhibits high fault tolerance and enables noiseless operation.

### 3.2. Boost Converter

A boost converter (also known as step-up converter) is one of the simplest types of switch-mode converters that can increase an input voltage. It raises the level of DC voltage from low to high while also decreasing the current from high to low. A boost converter consists of an inductor, a semiconductor switch, a diode, a capacitor and a load.

The principle of the boost converter is based on an inductor that resists changes in a current by either increasing or decreasing the energy stored in the inductor’s magnetic field. When the switch is closed, current flows through the inductor, which stores some energy by generating a magnetic field. When the switch is opened, a reduction in the output current occurs since load impedance is higher. This causes a reduction in the magnetic field to maintain the current for the load and a reversal in polarity, which increases the voltage to charge the capacitor through the diode. If the switch is cycled quickly enough, the inductor is not fully discharged between charging stages and creates a greater output voltage than input voltage [32,33].

The boost converter can function in either continuous mode or discontinuous mode. In continuous mode, the current through the inductor never falls to zero, whereas in the discontinuous mode, the current can fall to zero and, hence, affect output voltage. A major advantage of a boost converter is its high efficiency of up to 50% [34].

### 3.3. Temperature Difference between Soil and Air

Soil temperature varies depending on whether it is surface soil (depth = 0 cm) or a deeper layer of soil (depth > 0 cm), called underground soil. Underground soil temperature is affected by temperature changes in the near-surface air, which varies cyclically both in daily (short cycle) and yearly (long cycle) conditions [35]. The temperature of underground soil at a depth of a few tenths of a metre is affected mainly by the annual long cycle of air temperature changes and not the daily short cycle. The greater the soil depth, the greater the temperature difference between the air and the soil. Ikeda et al. [27] concluded that the temperature difference between near-surface air and shallow underground soil at a depth of 0.3 m is suitable for power generation. At a depth of 0.5 m, the daily temperature fluctuations are very small, and at a depth of 1 m, the temperature does not fluctuate over the course of a day [35].

## 4. Experiment

This section describes an experimental sensor module composed of a TEG, sensor, boost converter and a load. The remainder of this section provides input data and reference solution descriptions to evaluate the model.

**Figure 2:**
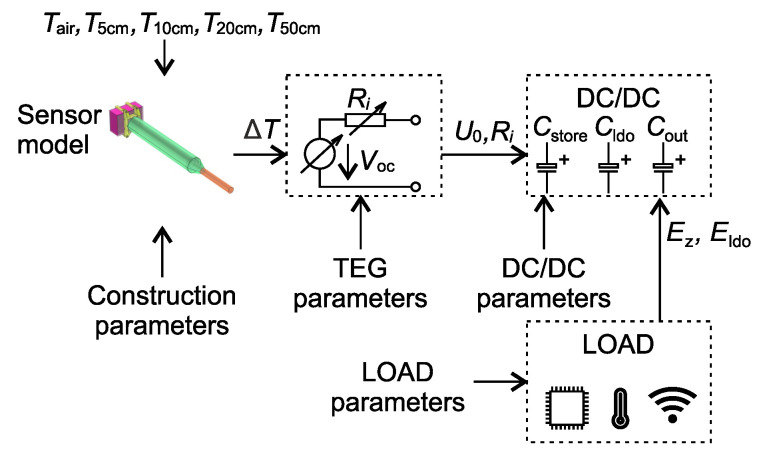
Block diagram of the simulation model. The sensor module supplies a DC/DC converter through the TEG module. The load simulates data transmission, control algorithm and data collection.

A scheme of the setup is shown in Figure 2. Each block represents a mathematical description of EWSN. These blocks are described below in the following subsections.

### 4.1. Input Data

The input data used in the experiment were obtained from the Czech Hydrometeorological Institute [36], which provides certified data. The founder of this organization is the Ministry of the Environment of Czech Republic. The data contained values of air temperature and near-surface soil temperature collected over a period of one year (2016). The air temperature values were measured at 10 min intervals in the Churanov area in Czech Republic. The soil temperature values were measured at the same location and times at depths of 0.05 m, 0.1 m, 0.2 m and 0.5 m. The Churanov Monitoring station in the Czech Republic is located at the coordinates 49.0683${}^{\circ }$ latitude, 13.615${}^{\circ }$ longitude and 1117.8 m elevation.

### 4.2. Sensor Simulation Model

This section describes the sensor simulation setup and sensor composition. For the purposes of implementation, the finite element method (FEM) was applied to simulate the temperature difference between the lower and upper surfaces of the TEG. The simulation model was created in Comsol Multiphysics 5.6 software with the Heat Transfer Module.

**Figure 3:**
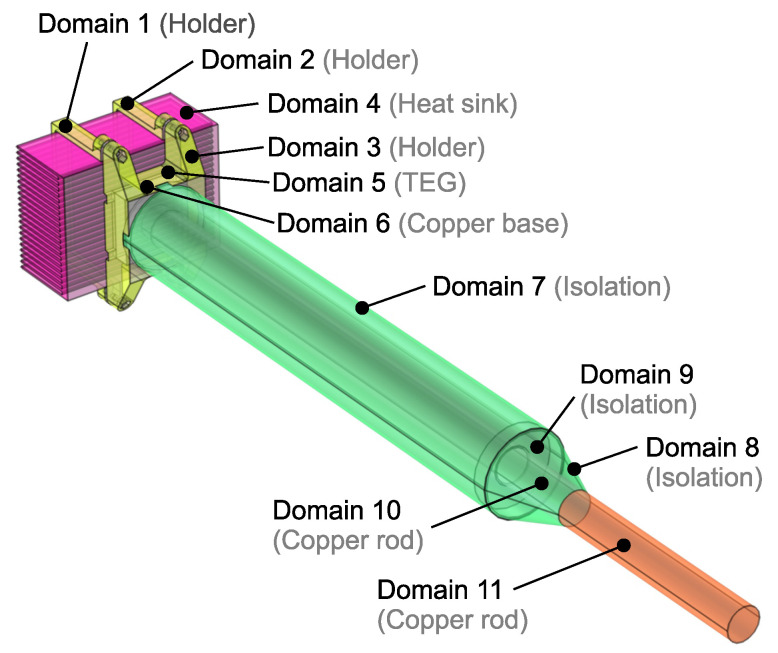
Sensor composition: Domain 1—Holder, left part; Domain 2—Holder, right part; Domain 3—Holder, lower part; Domain 4—Heat sink; Domain 5—TEG; Domain 6—Copper base; Domain 7—Isolation tube, main part; Domain 8—Isolation tube, lower conical part; Domain 9—Inner insulation fill; Domain 10—Copper rod, middle part; Domain 11—Copper rod, lower active part.

Figure 3 shows the composition of the sensor divided into eleven domains. The purple area depicts the heat sink, the yellow area represents a holder with the TEG and copper base, the green area represents the isolation tube and the orange area represents a copper rod.

The simulation procedure comprised several stages:

**1. Addition of a 3D component:** When a new 3D component is added to the Comsol model builder, the geometry must be defined. In this case, the geometry was imported from the original 3D model created in Autodesk Inventor Professional 2022. The transfer of geometry was performed using a STEP file. Some post-import actions were performed in order to adjust the geometry for simulation purposes. In particular, because of the different boundary conditions, the copper rod was divided into two sections with a workplane to form two separate domains (Domain 10; Domain 11). Suitable boundaries denoted “selections” in the Comsol notation for several of the domains were selected and grouped into four categories for further processing, and different boundary conditions were applied.

**2. Global definitions:** In this section of the Comsol Model Builder, additional functions were set for required purposes, i.e., an interpolation function to define the temperature profile in the soil, a function table containing measured air temperatures and a function table containing measured soil temperatures at several predefined depths (i.e., 5, 10, 20 and 50 cm).

**3. Addition of physics**: Due to the fact that the nature of the explored phenomenon is thermally spread in a solid mass, the Heat Transfer in Solids interface (ht) was used for this part of the simulation. This interface is generally used to model heat transfer in solids by conduction, convection or radiation.

**4. Definition of Materials:** The model includes several types of material that had to be defined for the purposes of the simulation. Table 4 lists the materials applied in the simulation from the built-in Comsol material library.

**5. Definition of the Heat Transfer problem:** In order to launch the simulation, the initial conditions had to be defined. In this case, by using the “Initial Values” context menu of the Heat Transfer interface, the initial value for the entire model (all domains) was set to 15 ${}^{\circ }$C. Using the “Temperature” context menu of the Heat Transfer interface, the next step defined the Dirichlet for the boundary conditions. Table 5 contains details of the conditions of the outer surface temperatures on the sensor model.

All outer surfaces above the ground were exposed to ambient air temperature. The outer surfaces of the isolation tube were exposed to the soil heat source according the interpolated temperature profile. Finally, the uncovered active end of the copper rod was exposed to the soil heat sources corresponding to a depth of 50 cm.

**6. Addition of a study:** Using “Add Study” from the main menu, a time-dependent study was added to reflect the dynamics of the explored process. Since the definition of the problem incorporated only one type of physical phenomenon (heat transfer in solids), specification of the vector of the output times where the solution would be sought was necessary. In this case, a range in seconds reflecting the entire period of the solution (1 year) was selected, the time intervals being the period between two air temperature measurements (soil temperature was measured once every hour).

**7. Post-processing (results)** Under the “Datasets” node in the Model Builder, the objects of interest (points and surfaces) where the results would be displayed were defined. Under the “Results” node in Model Builder, a 3D, 2D or 1D plot group according to the particular objects of interest can be added.

**Figure 4:**
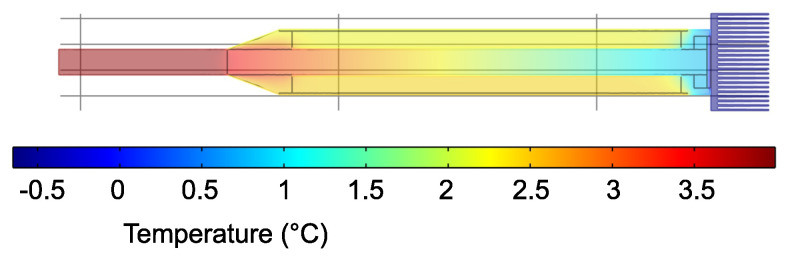
Model of the temperature distribution in the sensor. Red represents higher temperature, and blue represents lower temperature.

Figure 4 illustrates the temperature distribution in the sensor, showing temperature changes in the soil from the surface to a certain depth.

The relevance of the simulation is ensured by the fact that no user component, such as equation modification, and no custom parameters in physics and materials were used. In particular, in this case, used materials have great impacts on simulation relevance. In this simulation, all materials are defined in the COMSOL Material Library and exactly agrees with the materials that are designed for prototype devices. Therefore, the properties of the materials and, thus, the results of the simulation are guaranteed by the COMSOL supplier. More detailed information about COMSOL simulation and materials can be found in [37,38,39].

### 4.3. Thermoelectric Generator Block

**Figure 5:**
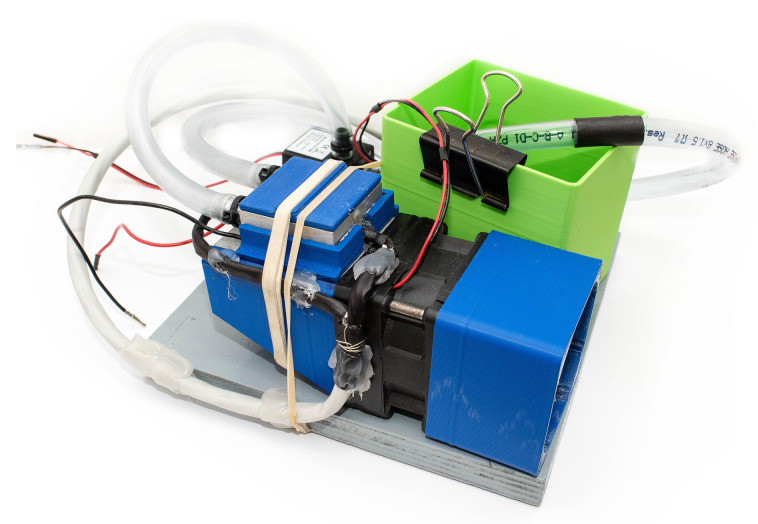
Experimental setup equipped by TEC1-12706. The fan cools the TEG from one side, and hot water is pumped onto the second side of the TEG.

A TEC1-12706 [40] module specified by the manufacturer for cooling was used for the experiment. This TEG can be exposed to various temperature differences to generate a suitable amount of electrical energy. The TEG’s properties were obtained by experimental measurement of current and voltage characteristics for each temperature difference. The experimental setup equipped by TEG is shown in Figure 5. This setup contains TEG itself, heating by water and cooling by an air. The hot water is pumped by an electrical pump from a small tank to one side of the TEG. On the second side, there is a heat sink with a fan in order to ensure heat dissipation. Moreover, two temperature sensors are placed on both sides of the TEG.

The open-circuit voltage $V_{\mathrm{oc}}$ and internal resistance $R_{i}$ were calculated for each temperature difference from these characteristics. The characteristics of the TEG model can be expressed according to Equation (2):

(2) $$V_{\mathrm{oc}},\;R_{i}=f\left(\Delta T\right),$$

where $V_{\mathrm{oc}}$ is the TEG’s open-circuit voltage, $R_{i}$ is the internal resistance and ΔT is temperature difference between the hot and a cold sides.

**Figure 6:**
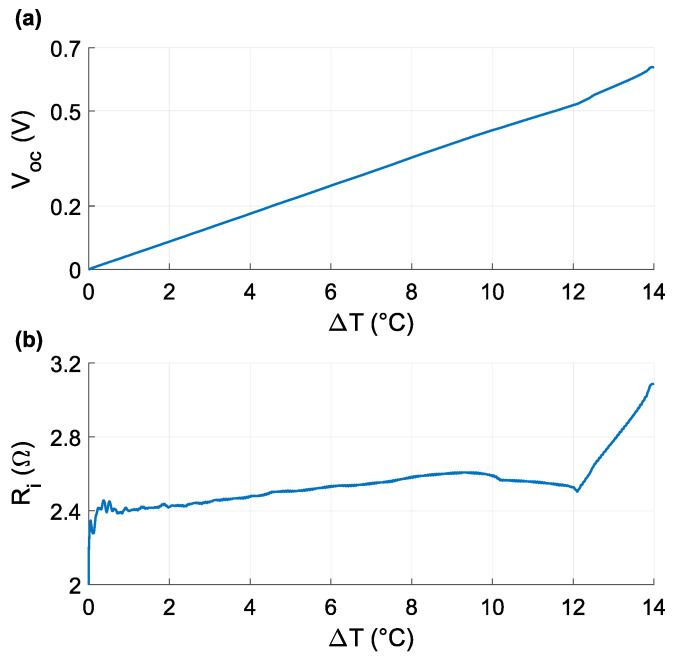
(**a**) Graph of open-circuit voltage $V_{\mathrm{oc}}$ according temperature difference ΔT; (**b**) graph of internal resistance $R_{i}$ according temperature difference ΔT.

Figure 6 graphs the $V_{\mathrm{oc}}\left(\Delta T\right)$ and $R_{i}\left(\Delta T\right)$ of the module according to temperature change. The open-circuit voltage demonstrates an approximately linear function, while the internal resistor value follows a mostly nonlinear function. The $V_{\mathrm{oc}}\left(\Delta T\right)$ and $R_{i}\left(\Delta T\right)$ values were used to determine a Thévenin-equivalent series circuit.

### 4.4. Boost Converter Block

TEG produces an open circuit voltage from tens to hundreds of millivolts, which is insufficient for the direct supply of electrical devices such microcontrollers or transmission modules. The voltage can be boosted by a DC/DC converter, for example, an LTC3109 module, which is a device dedicated to converting electrical energy from extremely low input voltage sources such as TEGs [41]. It allows operation with a voltage input as low as 30 mV and both input polarities. The LTC3109 module provides an output of 2.2 V for very low power systems and one extra output of 2.35–5 V [41].

**Figure 7:**
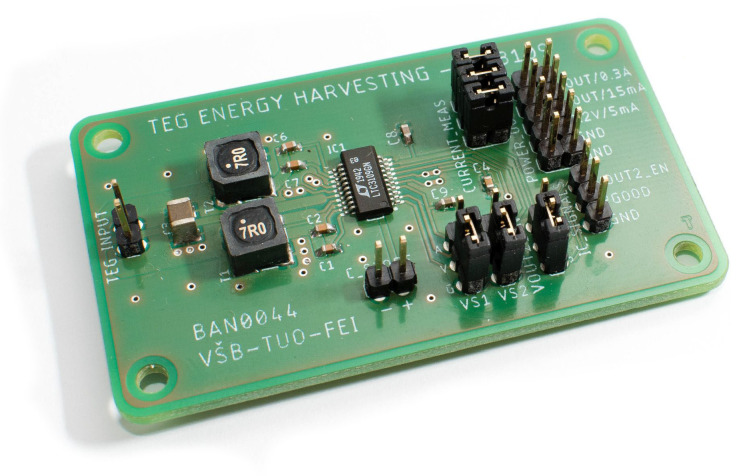
Experimental printed circuit board equipped by LTC3109 DC/DC converter.

The mathematical model of the DC/DC converter was designed with respect to the main physical aspects and basic functionality of the LTC3109. The functionality of the DC/DC converter was evaluated by measurement using experimental printed circuit layout boards, which are shown in Figure 7. This setup allows measuring voltage and current on the input and output pins and $V_{\mathrm{STORE}}$ as well. By using these parameters, the efficiency of DC/DC conversion could be evaluated. Moreover, the efficiency of charging and discharging of the supercapacitor could be measured.

A typical application circuit described in the LTC3109 datasheet uses three additional capacitors as energy sources in the case that input energy is in short supply. The capacitor $C_{\mathrm{LDO}}$ (typically 2.2 μF) stores the $V_{\mathrm{LDO}}$ output, and the capacitor $C_{\mathrm{OUT}}$ (typically 470 μF) is connected to the $V_{\mathrm{OUT}}$ pin. The supercapacitor $C_{\mathrm{STORE}}$ (1 F, 5.25 V) is able to supply output when input provides insufficient power. In the presented study, the DC/DC converter model was used to produce $V_{\mathrm{OUT}}=2.35\;V$ output voltage.

**Figure 8:**
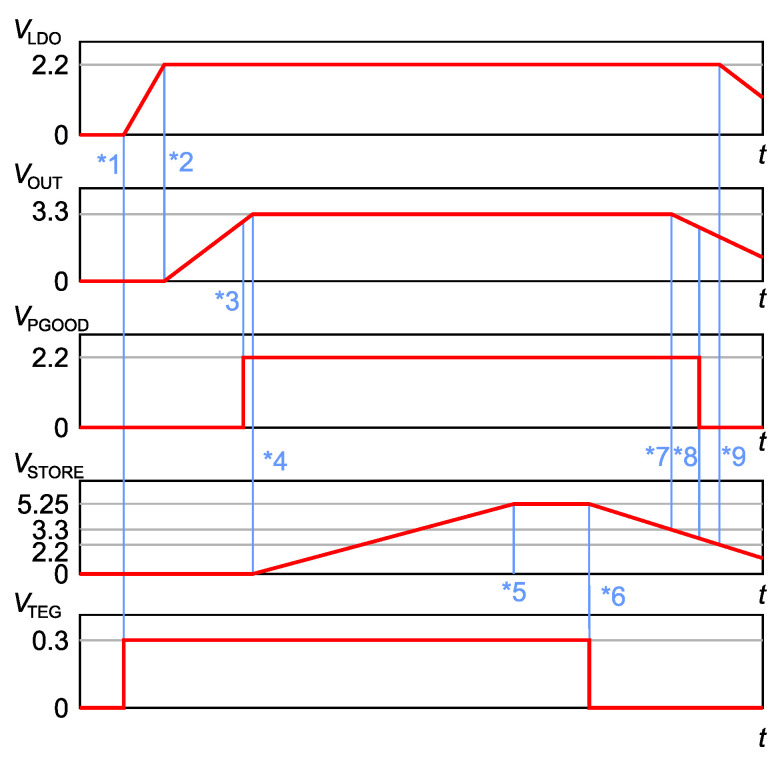
Output voltage sequences for input ($V_{\mathrm{TEG}}$), storage ($V_{\mathrm{STORE}}$), outputs ($V_{\mathrm{LDO}}$, $V_{\mathrm{OUT}}$) and status ($V_{\mathrm{PGOOD}}$).

The functionality of the boost DC/DC block is shown in Figure 8 as a demonstration of $V_{\mathrm{OUT}}=3.3\;V$ output voltage. This figure illustrates the dependency of the output voltages according to whether input power is present or absent. It also includes nine distinguishing indicators that are important in understanding the functionality of the LTC3109 module.

From the first indicator (*1), the input voltage ($V_{\mathrm{TEG}}$) proceeds from zero to 0.3 V and $C_{\mathrm{LDO}}$ begins to charge, with a consequent rise in $V_{\mathrm{LDO}}$. When $V_{\mathrm{LDO}}$ reaches 2.2 V, the output capacitor $C_{\mathrm{OUT}}$ begins to charge (indicator *2). The output capacitor $C_{\mathrm{OUT}}$ is directly connected to the $V_{\mathrm{OUT}}$ pin; therefore, the output voltage $V_{\mathrm{OUT}}$ corresponds to the $C_{\mathrm{OUT}}$ voltage. When $V_{\mathrm{OUT}}$ reaches 92.5% of nominal voltage (indicator *3), the power good (PGOOD) pin is activated. The storage capacitor $C_{\mathrm{STORE}}$ begins to charge once $V_{\mathrm{OUT}}$ reaches 100% of nominal voltage (indicator *4) and is fully charged when $V_{\mathrm{STORE}}$ is 5.25 V (indicator *5). The sequence tagged with indicator *6 shows how the LTC3109 functions with charged capacitors and no input power ($V_{\mathrm{TEG}}=0$). $C_{\mathrm{STORE}}$ is discharged by a load, and $V_{\mathrm{OUT}}$ holds nominal voltage until $V_{\mathrm{STORE}}$ drops (indicator *7) below $V_{\mathrm{OUT}}$ nominal voltage (3.3 V), and then $V_{\mathrm{OUT}}$ is equal to $V_{\mathrm{STORE}}$. When $V_{\mathrm{OUT}}$ drops below 91% of nominal voltage, the power good (PGOOD) pin drops (indicator *8). LDO output $V_{\mathrm{LDO}}$ maintains 2.2 V until $V_{\mathrm{STORE}}$ drops below 2.2 V, when $V_{\mathrm{LDO}}$ also starts falling (indicator *9).

A mathematical model of the TEG was used as an input DC/DC converter to provide $V_{\mathrm{oc}}$ and $R_{i}$ values. LTC3109 is able to optimize the power transfer from TEG; therefore, the ideal power transfer is assumed in this experiment. The input current to the DC/DC converter is calculated according to the following equation.

(3) $$I_{\mathrm{in}}=\frac{V_{\mathrm{oc}}}{2\cdot R_{i}}$$

The $V_{\mathrm{TEG}}$ voltage is calculated according to the following equation.

(4) $$V_{\mathrm{TEG}}=R_{i}\cdot I_{\mathrm{in}}$$

**Figure 9:**
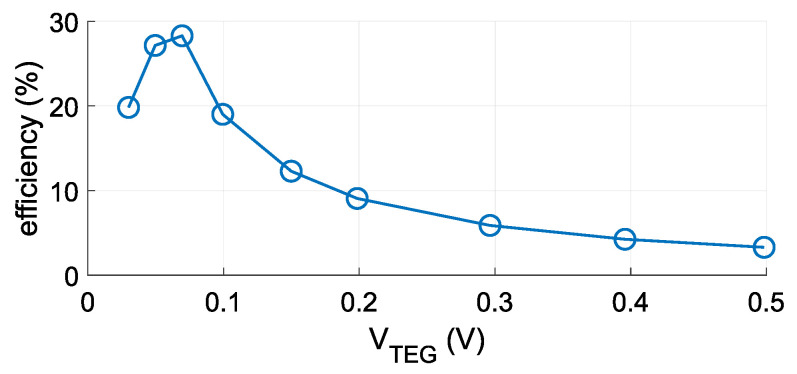
LTC3109 efficiency according to input voltage $V_{\mathrm{TEG}}$.

Figure 9 charts the efficiency of voltage conversion in the LTC3109 module. The harvested energy can be calculated with the equation:

(5) $$E_{\mathrm{Harvested}}=V_{\mathrm{TEG}}\cdot I_{\mathrm{in}}\cdot \eta \left(V_{\mathrm{TEG}}\right)\cdot \Delta t,$$

where $E_{\mathrm{Harvested}}$ is the harvested energy, $\eta (V_{\mathrm{TEG}})$ is the efficiency depending on $V_{\mathrm{TEG}}$ and Δt is the time between two simulation steps.

The harvested energy $E_{\mathrm{Harvested}}$ is used to supply a load and to charge the capacitors. The general equation that describes the relationship between capacitor voltage and energy is as follows:

(6) $$E=\frac{1}{2}\cdot C\cdot V^{2},$$

where *E* is the energy stored in the capacitor, *C* is the capacitance and *V* is the capacitor voltage. Equation (6) allows the calculation of the capacitor voltage according the current amount of energy stored in the capacitor.

The self-discharging model is based on the parameters of the supercapacitor KW-5R5C105-R [42]. The supercapacitor’s datasheet specifies that the initial voltage drops to 70% after 2000 h; therefore, in one simulation step, the supercapacitor voltage decreases by approximately 0.002972% and can be described as follows:

(7) $$E\left(k\right)=E\left(k-1\right)\cdot 29.72\cdot 10^{-6},$$

where *E* is supercapacitor energy, and *k* is simulation step.

The simulation of boost converter block also includes the charging and discharging efficiency of the supercapacitor. The calculation of efficiency is based on power dissipation on serial resistor $R_{\mathrm{ESR}}$ = 30 Ω [42]. The efficiency can be calculated as follows:

(8) $$\eta =\frac{P}{P+R_{\mathrm{ESR}}\cdot I_{C}^{2}},$$

where *P* is charge/discharge power, $I_{C}$ is capacitor current and η is the efficiency.

### 4.5. Load Block

The theoretical model device contains a power source, a microcontroller, an environmental sensor, non-volatile memory and a wireless communications interface. The microcontroller was selected specifically for low power consumption. Moreover, a modern, 32-bit device with a wide range of integrated peripherals is desirable for use. An NXP KL25Z [43] device based on an ARM Cortex-M0+ based microcontroller was also selected because it fulfills the requirements of the architecture and provides a number of low power modes that allow finetuning the energy profile.

An environmental sensor serves as a data source. It should be noted that no particular sensor is prescribed for the defined task, but a sensor of the type that is expected in an advanced design is still desirable; a Bosch BME688 4-in-1 sensor [44] was, therefore, selected. This device was able to measure ambient temperature, air humidity and atmospheric pressure, and the integrated gas sensor was able to detect volatile organic compounds (VOCs), volatile sulphur compounds (VSCs) and various other gases. The BME688 was connected to the microcontroller via the I2C bus.

Generally speaking, data measurement and data transmission are two asynchronous operations. The data transmission channel may not be available immediately when measurement is complete, although it may become ready independently of the measurement. A memory buffer is required to synchronize these two operations; a 24CW1280 EEPROM [45] was, therefore, selected, although FRAM technology was also considered. FRAM memory has many advantages, especially a fast write time and a large number of write cycles, but it was unsuitable for the model device since it runs with a voltage as low as 1.8 V. Unfortunately, no FRAM devices were available that could function with a voltage this low.

A LoRaWAN was considered a viable communications solution since it is one of the most popular communications solutions for IoT devices today; a Semtech SX1261 [46] LoRa transceiver was, therefore, selected as a communications link. The SX1261 was connected to the microcontroller via the SPI bus. LoRaWAN technology offers three communication classes (indicated as class A, B and C) that cover various use cases. Class A offers the best energy saving mode but only very limited downstream capabilities; class C provides a continuous downstream channel but is the most energy demanding since the modem is permanently powered.

**Figure 10:**
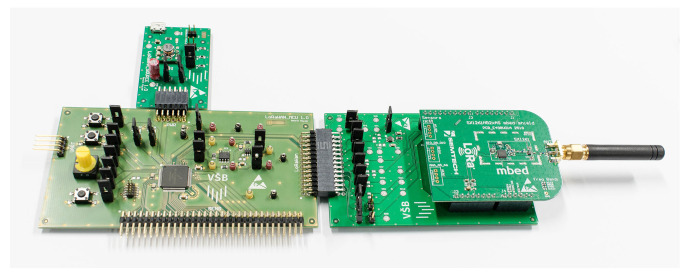
Experimental setup with MCU, LoRaWAN module and energy source.

Table 6 lists the parameters for power consumption of the simulated peripherals and their modes. The parameters were evaluated by using a LoRaWAN experimental setup depicted in Figure 10. The setup consist of semtech LoRa module, development board for LoRaWAN measurement, base board with MCU and power supply board. All boards include several test points that allows measuring voltage and current.

The experiment is based on switching two modes represented by sleep and run. If the device is in sleep mode, unused peripherals are deactivated, and the MCU is in VLPS. The required energy is calculated according to Equation (9).

(9) $$E_{\mathrm{SLEEP}}=P_{\mathrm{SLEEP}}\cdot \Delta t$$

If run mode is active, the total consumption is composed of peripheral consumption (Equation (10)).

(10) $$E_{\mathrm{RUN}}=P_{\mathrm{MCU}}\cdot t_{\mathrm{CYCLE}}+E_{\mathrm{MEM}}+E_{\mathrm{MEA}}+E_{\mathrm{TX}}+P_{\mathrm{SLEEP}}\cdot \left(\Delta t-t_{\mathrm{CYCLE}}\right)$$

From the technical documentation describing individual components, the power profile of the model device was then estimated. This estimation may not always be entirely accurate: For example, data caching may not be necessary because the data transmission channel is either immediately available or a measurement operation may require more time than expected. The estimation considered a variety of possible scenarios but discarded rare events such as data measurement failure or network provisioning operations performed on the wireless interface.

The power profile of the model device has two fundamental states: idle or operational. When the model device is idle, the remainder of the model device powers off. The only powered component in this mode is the MCU, but it does not execute instructions (in the MCU’s technical documentation, this mode is called Very Low Leakage Sleep 1, VLLS1). VLLS1 mode is the lowest power mode in which the real-time clock (RTC) circuit is operational. This is an important factor since the RTC must wake up the CPU at a preset time. The current during VLLS1 mode (including the current of the RTC) is estimated to be in the range of 0.973 to 16.08 μA [43]. The relatively wide margin is caused by the current’s heavy dependence on the ambient temperature. When the MCU exits sleep mode, a program is executed. Due to the fact that the model device has a low power character, the lowest power execution mode is selected. In the KL25Z MCU, this mode is called a Very Low Power Run, VLPR. Power consumption is greatly reduced in the VLPR mode, but the current may vary greatly. Different on-chip MCU peripherals can be enabled or disabled according to the needs of the application, but the individual machine instructions comprising the application draw different amounts of power. These are major factors that cause current variability in VLPR mode; the current is estimated to fall in the range of 171 to 777 μA [43].

The power-off state of the environmental sensor, EEPROM memory and LoRa transceiver is attained by using dedicated power switches. Individual devices are powered on and off sequentially. First, the BME688 sensor performs a measurement. According to the technical documentation [44], a measurement may take up 10.8 s, and the current during measurement is approximately 3.9 mA. One page of data is then written to the 24CW1280 EEPROM. This operation requires 5 ms, and the current is 1 mA [45]. After EEPROM access, a wireless transmission via the LoRaWAN network is performed. The class A communications profile is applied, and communication opens with a transmission burst, which takes around 500 ms with a current of up to 48 mA. Two similar receive operations are then attempted, each consisting of a 250 ms delay (*I* = 2.1 mA) followed by a 250 ms receive window stage (*I* = 8.2 mA) [45].

### 4.6. Reference Solutions

To evaluate the proposed TEG-powered module that harvests energy according to an air and near-surface ΔT, three reference solutions were applied. A comparison of the individual solutions is summarized in Table 2.

Ikeda et al. (R1) developed a sensor prototype driven by a TEG (KELK Ltd. KTGM 199-2, Hiratsuka, Japan) that was able to harvest on average more than 100 μW at a ΔT of 2–35 ${}^{\circ }$C between the air and underground soil at a depth of 30 cm. [27]. Huang et al. (R2) used a TEG (TG12-6-02 from Marlow Industries) to generate electricity at a ΔT of 0–26.5 ${}^{\circ }$C at a depth of 0.3–3.0 m. The results showed the feasibility of powering wireless sensors with a power of 76–335 μW [28]. Wang et al. (R3) used a hybrid system (photovoltaics and a TG12-8 from Marlow Industries) to provide a stable WSN output at a ΔT of 0–25 ${}^{\circ }$C and depth of 2.5 m. The TEG produced an average output power of 200–324 μW [29].

By evaluating and comparing reference solutions, the following pros and cons were found. The advantage of the R1 article is a research impact by realizing a battery-free sensor. The advantage of the R2 article is proof of TEG powering wireless sensors in remote areas by performing experimental monitoring of devices for 6 months. The advantage of the article R3 includes providing stable power to WSNs by using a hybrid energy harvesting system.

### 4.7. Performance Evaluation

The simulation was evaluated by several criteria and performance characteristics. At first, the simulation was evaluated in terms of successfully (complete) cycles, missed cycles and the ratio. A missed cycle is a period during which transmission was required but energy was insufficient. The ratio represents the percentage of successful cycles and total periods. The ratio can be calculated as follows.

(11) $$\mathrm{Ratio}=\frac{\mathrm{Complete}}{\mathrm{Complete}+\mathrm{Missed}}\cdot 100\%$$

The next evaluation parameter is maximal delay (Max. delay). The maximum delay is the maximum time of system outage caused by insufficient energy in the harvesting module. It is calculate as a maximal time between consecutive missed cycles.

The simulation is also evaluated in terms of energy usage. The unused energy $E_{U}$ is sum of energy when the supercapacitor is fully charged and the load does not utilize all produced energy. The unused energy is expressed as the ratio between the sum of unused energy and sum of produced energy.

The simulation was performed for various load properties and time to discharge (TTD), and overheads were calculated. TTD was calculated as follows:

(12) $$E_{\mathrm{store}}=\frac{1}{2}C_{\mathrm{store}}\cdot V_{\mathrm{store}\;\max}^{2}-\frac{1}{2}C_{\mathrm{store}}\cdot V_{\mathrm{store}\;\min}^{2},$$

where $E_{\mathrm{store}}$ denotes usable energy, $C_{\mathrm{store}}$ is the capacity of the supercapacitor, $V_{\mathrm{store}\max}$ = 5.25 V denotes maximum $C_{\mathrm{store}}$ voltage and $V_{\mathrm{store}\min}$ = 2.35 V is the minimal $C_{\mathrm{store}}$ voltage:

(13) $$\mathrm{TTD}=\frac{E_{\mathrm{store}}}{E_{\mathrm{run}}+E_{\mathrm{sleep}}}\cdot \Delta T,$$

where TTD is time to discharge parameter, $E_{\mathrm{run}}$ is required energy for one cycle, $E_{\mathrm{sleep}}$ is required energy for one cycle of sleep and ΔT is the simulation period.

Overheads are defined as ratio between sleep mode energy $E_{\mathrm{sleep}}$, and total energy consumption and can be calculated as follows.

(14) $$\mathrm{Overheads}=\frac{E_{\mathrm{sleep}}}{E_{\mathrm{sleep}}+E_{\mathrm{run}}}\cdot 100\%$$

## 5. Results

**Figure 11:**
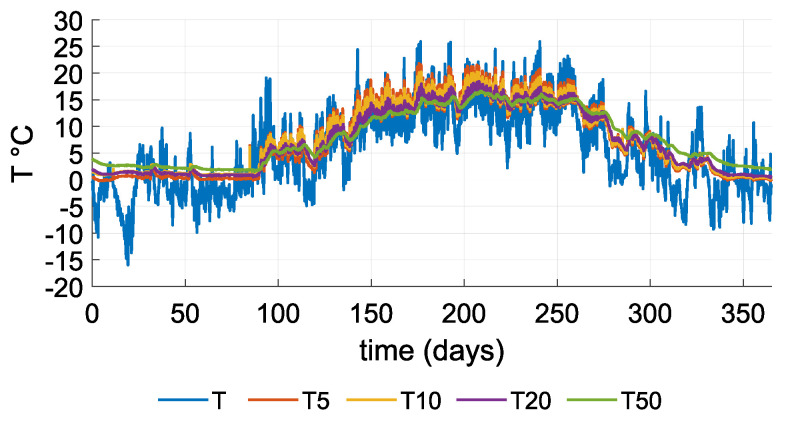
Input data: T is air temperature, T5 is soil temperature at 5 cm, T10 is soil temperature at 10 cm, T20 is soil temperature at 20 cm and T50 is soil temperature at 50 cm.

The experimental section described a simulation performed using historical air and soil temperature data. Figure 11 shows the air and soil temperatures at several depths over the course of a year. Variation in the soil temperature decreased with soil depth; therefore, the variation in difference between air temperature and temperature in deeper soil increased.

Soil temperature and air temperature are inputs for the sensor model to determine temperature difference (ΔT). The dataset of temperature differences was calculated as an output of the FEM simulation.

**Figure 12:**
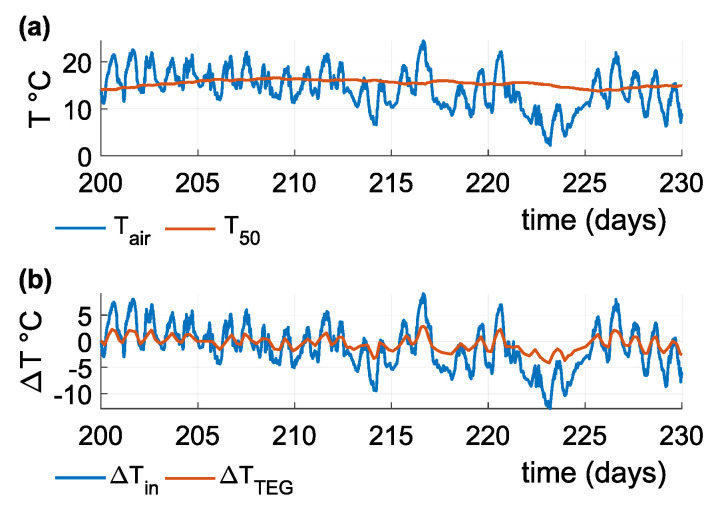
(**a**) Air and soil temperatures at a of depth 50 cm during summer, showing a large oscillation of air temperature around soil temperature; (**b**) temperature difference at the sensor input and temperature difference on the TEG.

**Figure 13:**
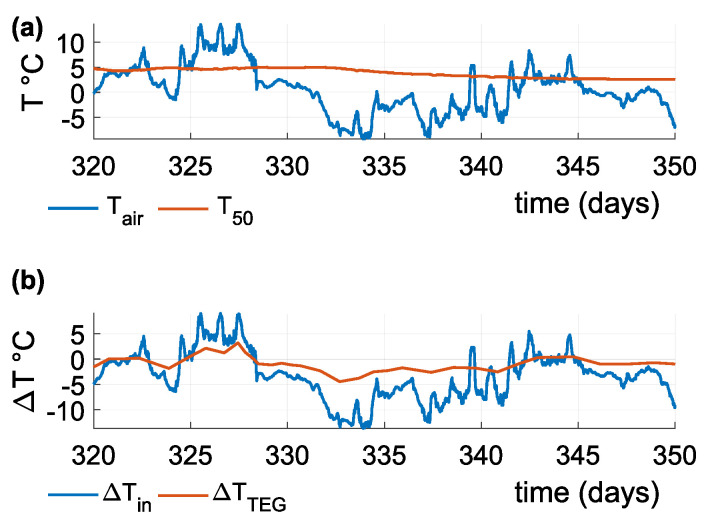
(**a**) Air and soil temperatures at a depth of 50 cm during winter. Air temperature differs from the soil temperature and creates a stable temperature difference; (**b**) temperature difference at the sensor input and temperature difference on the TEG.

The active surface of the heat conductor is located at a depth of 50 cm. Figure 12a and Figure 13a show the input temperatures acting on the active surface of the heat conductor and the heat sink’s surface. Figure 12b and Figure 13b show the difference between air and soil temperature, and the temperature difference on the TEG. Figure 12 charts measurements for the summer season: Air temperature oscillates around soil temperature, and the temperature difference decreases below the absolute value of one degree Celsius, resulting in the DC/DC converter not being able to function as required. Figure 13 charts measurements for the winter season: For most of the time, air temperature is either below or above soil temperature and provides a sufficient temperature difference. Air temperature varies with soil temperature during significant changes in the weather.

**Figure 14:**
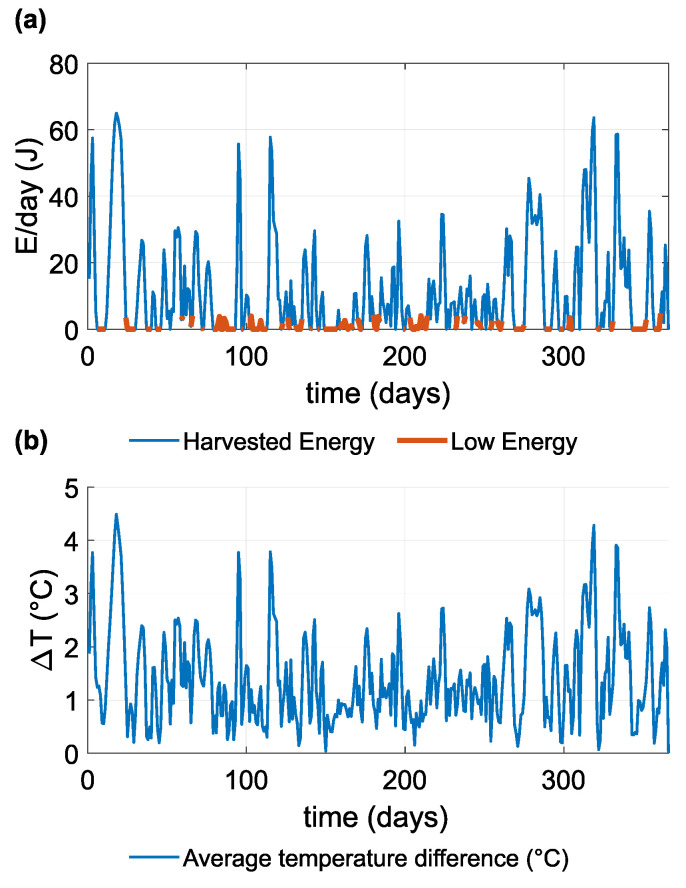
(**a**) Graph of daily harvested energy over the course of one year, where the quantity of harvested energy less than the median of harvested energy (4.82 J) is indicated as Low Energy; (**b**) graph of the average daily temperature difference on the TEG.

Figure 14 shows the daily average temperature differences (absolute values) on the TEG and daily totals of harvested energy. The quantity of harvested energy is estimated by the TEG model and is highly dependent on the temperature difference between the cold and hot sides. Although the temperature difference between soil and air reaches up to 12 ${}^{\circ }$C (Figure 11) the temperature difference on the surface of the TEG is not greater than 4 ${}^{\circ }$C. Even so, the temperature difference on the TEG is mostly less than 4 ${}^{\circ }$C, thereby allowing the DC/DC converter to function and harvest a small quantity of energy. The DC/DC converter is able to function as required when the input voltage is greater than 30 mV, which corresponds to a temperature difference of approximately 1 ${}^{\circ }$C. Figure 14 also shows the quantity of harvested energy over the course of a day, the maximum quantity being around 60 J, although the median is only 4.82 J.

The simulation modeled the efficiency of DC/DC converter and also efficiency of charging and discharging os supercapacitor $C_{\mathrm{STRORE}}$. The efficiency of the DC/DC converter is defined by a datasheet (see Figure 9). The charging efficiency calculation is based on power dissipation on $R_{\mathrm{ESR}}$. Higher charging current causes lower charging efficiency. Assume the worst case, which is calculated as the maximum power from TEG used for supercapacitor charging (no energy is used by load). The supercapacitor is charged when $V_{\mathrm{out}}$ reaches 2.35 V, so charging efficiency in these conditions is at least 99.7%. The discharging efficiency is calculated for two cases: sleep and run mode. Assume that the supercapacitor volatage is approximately 2.35 V; thus, the output current is equal to the supercapacitor current (worst case). In this case, the efficiency is 99.9%. For the run mode, it is assumed that the average output current is 5.8 mA. For a minimal supercapacitor voltage of 2.35 V, the discharging efficiency is 93.1%, and for a maximal supercapacitor voltage of 5.25 V, the discharge efficiency is 98.5%.

**Figure 15:**
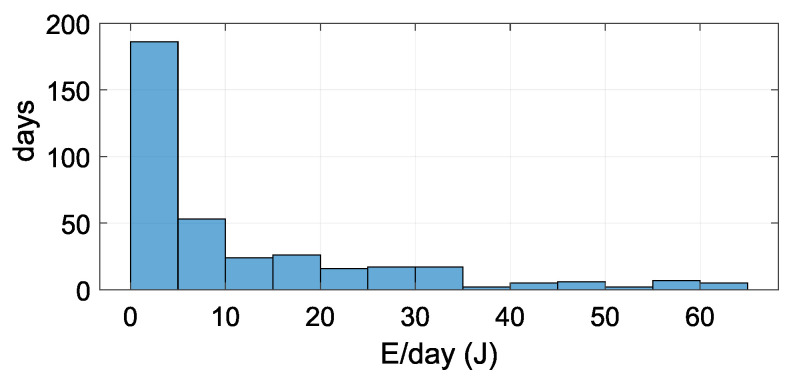
Histogram of daily harvested energy over the course of one year. On most days of the year, the quantity of energy harvested was between 0 and 5 J.

Figure 15 shows the histogram of daily harvested energy over the course of one year. On more than 50% (186 from 365) of days, the DC/DC converter harvests less than 5 J of energy. Five joules of harvested energy is equivalent to an electrical current of 24.6 μA at a voltage of 2.35 V per day.

Table 7 shows the statistical parameters of daily harvested energy, daily average electrical power and calculated output current for the specified output voltage. The daily harvested energy at the 25th percentile was zero, indicating at least 91 days of the year when it was not possible to harvest any energy.

Harvested energy is used to power the load block, which consist of a microcontroller, a transmission module and a data collection module. The load block modes are controlled by the MCU, which switches between run and sleep modes. Implementation of this finite state machine allows several combinations of duty cycles. A simulation of the MCU duty cycle was executed for six cases. The load model measured and transmitted data simultaneously according to a defined period only when the power good pin was active. The simulation was performed for three period settings (10, 60 and 240 min) and two sleep current settings (1.65 μA and 15.3 μA).

Table 8 documents the simulation results for complete and missed cycles over the course of one year. A complete cycle is an executed cycle, while a missed cycle is a cycle when run mode should be enabled but the available energy is insufficient. The ratio parameter represents the ratio of complete and total (complete + missed) cycles. The maximum delay is the maximum time of system outage caused by insufficient energy in the harvesting module. The unused energy parameter represents the ratio of unused energy and total harvested energy over the course of one year. Case 1 and Case 4 represent a short operation period; in these cases, the total number of complete or missed cycles reached the highest values. By contrast, Case 3 demonstrates the opposite, missing only 35 cycles over the course of the year. The EWSN functioned as required for 98.4% of the time, representing only 35 missed cycles out of a total 2190. In this case, the maximum delay was 2 days, representing 12 consecutive missed cycles. Other configurations experienced significantly larger maximum delays in the range of 7.3–10 days.

The time to discharge (TTD) parameter represents operational time without input energy from the TEG and a fully charged supercapacitor. The configurations with a short period (one and four) did not store additional energy, and during periods of insufficient input energy, energy was fully drained from storage after approximately 0.4 days, and the device stalled. Cases with long periods required lower quantities of energy. Case 3 was able to operate up to 7.88 days without input energy. The disadvantage of configurations with a long operational mode is that a large quantity of energy is used for sleep. The overhead represents the ratio of sleep consumption for two operational modes and the total energy consumption for one operational mode and sleep. Case 1 demonstrated the lowest overhead. Case 1 (1.2% overhead) applied a short sleep period and small sleep current. Cases 2, 3 and 4 demonstrated acceptable overheads of less than 25%. Cases 5 and 6 demonstrated relatively high overheads. Case 6 consumed 72.8% of energy in sleep mode. Table 8 also lists figures for unused energy $E_{U}$. Case 4 attained the highest power consumption, using 85.6% of harvested energy but missing 66% of transmission periods. Case 3 produced the lowest power consumption, using only 13.6% of harvested energy and missing only 1.6% of cycles.

**Figure 16:**
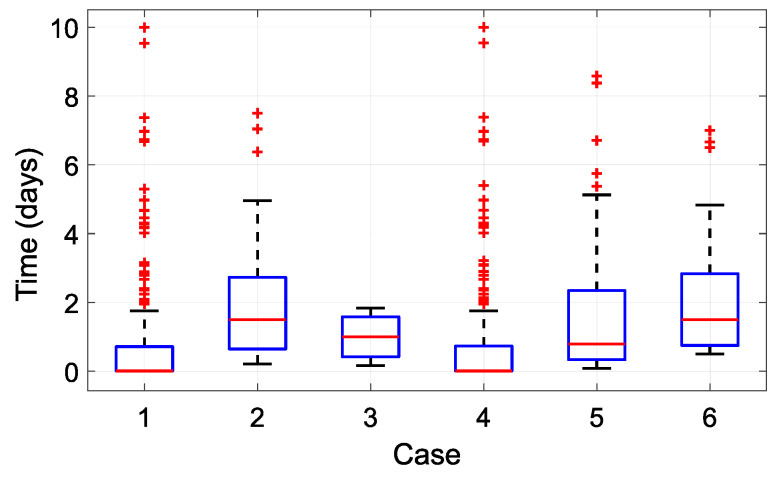
Boxplots of times between the first and last missed cycles in each configuration.

Boxplots of the times between the first and last missed cycles are shown in Figure 16. Cases 1 and 4 represent configurations with the shortest default operational period. This scenario uses energy immediately, and no stored energy is available for situations when a lack of input energy occurs. The boxplot outliers represent situations of long-term energy outages. Cases 1 and 4 both have similar statistical parameters since power is instantly dissipated as a result of power consumption having minimal effect during sleep mode.

The results of the 60-minute interval in Case 2 indicate very low operational performance due to numerous outages and a median delay time of 1.5 days. Case 3 demonstrated the most suitable results. It is a very stable solution, with 50% of the times between the first and the last missed cycles falling in the range 0.42–1.58.

The scenarios with high sleep power consumption (Cases 5 and 6) produced results different from Cases 2 and 3, revealing a significant effect from sleep current. Case 5 demonstrated low performance operation, similarly to Case 2. In Case 6, sleep current had a major effect, resulting in low performance compared to the 60-minute configuration (Case 5).

**Figure 17:**
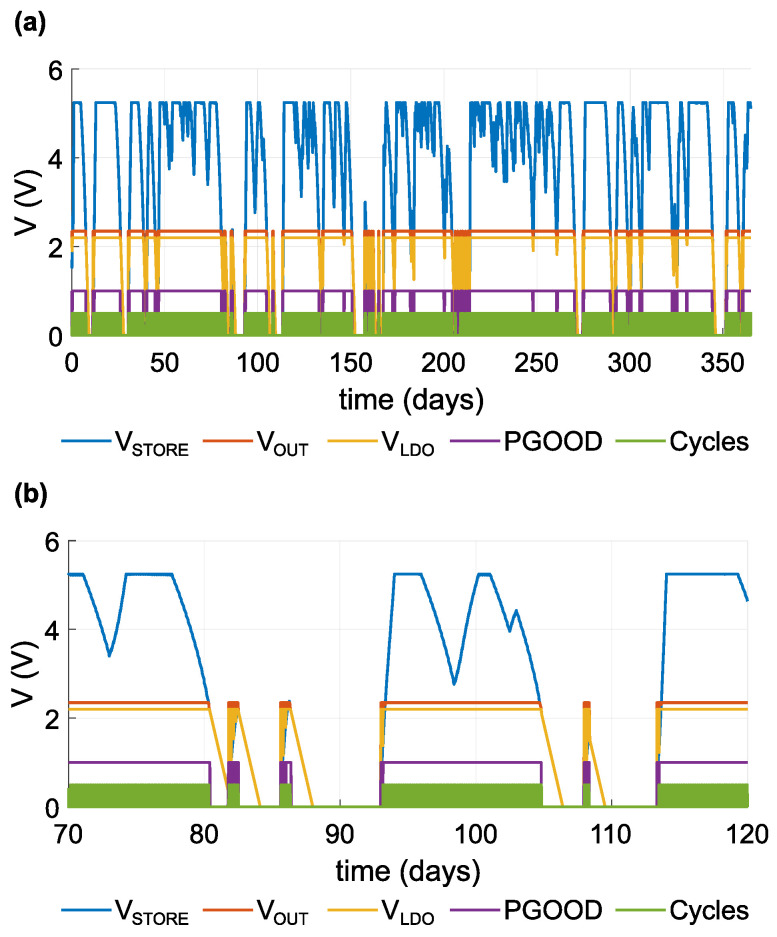
Graph of voltage levels; case $I_{\mathrm{SLEEP}}=15.3\;\mu A$, *T* = 4 h. (**a**) One year; (**b**) detail of days 70–120.

**Figure 18:**
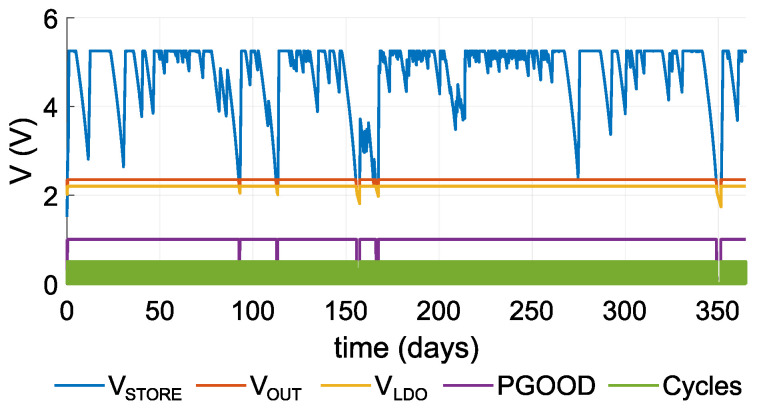
Graph of voltage levels; case $I_{\mathrm{SLEEP}}=1.65\;\mu A$, *T* = 4 h.

Figure 17 and Figure 18 chart the progress of the simulation. Figure 17 shows the EWSN configuration with a sleep current of 15.3 μA and 4-h period. Figure 17a displays the simulation results for the course of a year; Figure 17b provides a detailed view of a part of this period. The figures graph the simulated voltage parameters on the DC/DC converter’s output. Voltage $V_{\mathrm{STORE}}$ reflects the current status of the supercapacitor, with a maximum value of 5.25 V. $V_{\mathrm{OUT}}$ and $V_{\mathrm{LDO}}$ are DC/DC outputs, and PGOOD is the output pin that indicates that a nominal voltage of $V_{\mathrm{OUT}}$ is present. The cycle curve illustrates an active operational mode.

It is interesting that the EWSN functions are suitable as long as $V_{\mathrm{STORE}}$ maintains 2.35–5.25 V. If $V_{\mathrm{STORE}}$ falls below the 2.35 V threshold, there will not be sufficient energy availble to supply the EWSN. This range also represents a safe range in which the supercapacitor is able to supply the EWSN when no energy is available from the input. The $V_{\mathrm{STORE}}$ voltage is limited to 5.25 V. If the supercapacitor attains this value, no more storage is available for incoming energy, and the EWSN will not use all the input.

The results in the graphs indicate that the EWSN is able to function without input energy when the supercapacitor is charged, i.e., without any additional input energy, the EWSN in Case 3 operated for up to 7.88 days (Figure 18), and the EWSN in Case 6 operated for up to 2.76 days (Figure 17).

## 6. Discussion

The results stimulate several interesting areas of discussion, one area being a comparison of the experimental results to reference solutions [27,28,29]. The results indicate a daily average electrical power in the range 0–320.11 μW, which is directly comparable to the power generated by the TEGs listed in Table 2. The power ranges of the reference solutions are larger because of the TEG harvesting conditions and especially because of a larger range in the input temperature difference. The reference articles also describe infield experiments that bring facts about output power changes according to deployment location.

Another notable feature is the sensor module’s design, particularly the sensor’s length and ability to be deployed in a specific environment. The sensor’s length affects the temperature difference on the cold and hot sides of the TEG built into the module, corresponding to a quantity of generated energy. Ikeda et al. [27] concluded that the temperature difference between near-surface air and shallow underground soil at a depth of 0.3 m can be used to generate power. Jury et al. [35] indicated that at a depth of 0.5 m in soil, the daily temperature fluctuations are very small and that at a depth of 1 m, the temperature does not change over the course of a day. Applying these findings, we simulated the temperature data at a depth of 0.5 m. The module can be deployed at various locations in diverse climates. The temperature of soil at a depth of a few units of tenths of a metre is affected mainly by the longer annual cycle of changes in air temperature and not by the shorter daily cycles. The module can, therefore, be located in any geographical area in the world without significantly affecting the principle of harvesting energy from the TEG used to power an IoT device that monitors environmental parameters.

Management of available energy for the module also presented challenges. Energy harvested from a TEG is boosted by a step-up (boost) DC/DC converter designed specifically for thermoelectric energy harvesting. The DC/DC converter (LTC3109) used in the experiment was most effective when operating with small temperature differences (above 1 ${}^{\circ }$C). At a temperature of 2 ${}^{\circ }$C, the efficiency of the converter declined sharply. Future circuit designs by the manufacturer would require improvements to this drop in efficiency in the device. It is also worth considering the use of another type of DC/DC converter, which is equally suitable for harvesting thermoelectric energy when such an electronic component becomes available on the market. The stability of each simulated configuration with regard to sleep consumption and time to discharge is also an interesting question (i.e., operation with a minimum of outages). The results showed that the device is the most stable if the operating period is long (240 min) and the sleep current is low (set to 1.65 μA). A trade-off between sleep consumption and the operating period parameters may be required to minimize sleep consumption and energy wastage. The technology presents a broad area of application in smart energy management strategies based on machine learning.

Experiments have demonstrated and verified that TEGs can be used as a suitable power source for maintenance-free EWSN devices without batteries. EWSN devices are equipped with MCUs, measurement sensors and transmission modules such as LoRaWAN modules. These devices function in duty cycle modes but are inactive (sleep mode) most of the time. This configuration allows the device to harvest sufficient energy for measurement and data transmission. The results of the presented study show that the experimental device suffered delays caused by a temporary lack of energy in the energy harvesting subsystem. In the case of a fixed duty cycle, the delays were only directly dependent on the incoming energy. Again, the technology presents a broad area of applications in smart energy management strategies based on machine learning.

## 7. Conclusions and Future Work

The article presented a simulation of an environment-monitoring IoT device powered by a TEG which exploits the differences in air and soil temperature. The device included a sensor, a TEG, a DC/DC boost converter and a load. The sensor module was used to power a DC/DC converter from a TEG, while the load simulated data transmission, a control algorithm and data collection. The simulation represented a mathematical description of an EWSN and applied historical air and soil temperature data. The energy harvested from the TEG was used to power a load block controlled by an MCU to operate in two modes (sleep and active). The MCU operation cycle was simulated in six configurations with different settings for different periods (10, 60 and 240 min) and sleep currents (1.65 μA and 15.3 μA). The results showed that 0 to 65 J of energy could be harvested daily. The design of the EWSN load module included an LTC3109 DC/DC converter that operated under defined conditions. The device’s capacitor stored energy when insufficient energy was produced by the TEG.

Future work with this technology can be applied in several areas. One interesting area is optimization of the sensor’s construction itself. In the present configuration, the sensor’s weight, dimensions and material costs, especially for copper, are not optimal. The aim of future studies may involve reducing the dimensions of the sensor while maintaining sufficient input energy from the TEG. Another area involves optimization of the EWSN hardware, for example, selection of a superior storage capacitor, improved DC/DC converter design and embedding all components on an electronic board (MCU, sensors, data storage, IoT communication modules, etc.). One very interesting area is in designing energy management strategies to optimize the stability of the EWSN node and to specify its behaviour. Broad scope exists in implementing various machine learning methods to manage energy consumption when sufficient energy is available or the storage of energy in a local supercapacitor when a lack of energy is indicated. In terms of practical implementation, the authors of the presented study are now testing the presented solution in field conditions and are aiming to publish the results of long-term testing (months or a year).

## Figures and Tables

**Table 1:** Summary of TEG used in outdoor applications.

Author, Source	TEG/TEG Material	Purpose
Datta et al. [20]	TXL-287-03Z	LED lights for signage, illumination of roadways
Tahami et al. [21]	SP1848	LED traffic lights and wireless sensors
Lan et al. [22]	GM250-127-28-10	Wireless sensor node
Wang et al. [23]	$Bi_{2}Te_{3}$	Temperature sensor
Priya et al. [24]	TGM-127-1.4-2.5	Wearable biomedical IoT node
Praveena et al. [25]	$BhTe_{3}$, $Sb_{2}Te_{3}$	IoT location and temperature sensors
Seyoum et al. [26]	926-1192-ND	Temperature sensors and low power RF chips

**Table 2:** Summary of TEG reference solutions.

Label	Author, Source	TEG	DC/DC Convertor	Soil Depth (m)	ΔT (${}^{\circ }$C)	Power (μW)
R1	Ikeda et al. [27]	KTGM 199-2	LTC3109	0.3	2–35	94–369
R2	Huang et al. [28]	TG12-6-02	–	0.3–3.0	0–26.5	76–335
R3	Wang et al. [29]	TG12-8	LTC4071	2.5	3–25	200–324

**Table 4:** Materials applied from the Comsol material library to the various domains.

Material	Selection
Aluminium	Domain 4
Bismuth telluride	Domain 5
Copper	Domains 6,10,11
Polypropylene	Domains 7,8
Polyurethane	Domain 9
Polylactic acid, PLA	Domains 1,2,3

**Table 5:** Outer surface temperatures on the sensor model.

Category (Selections)	Description
heat_sink_surfaces	Outer surfaces of the heat sink (Domain 4) exposed to ambient temperature (air temperature)
holder_surfaces	Outer surfaces of the holder (Domains 1, 2, 3) exposed to ambient temperature (air temperature)
isolation_tube_surfaces	Outer surfaces of the isolation tube (Domains 7, 8) exposed to ambient temperature (soil thermal heat source approximated by linear interpolation of the temperatures at 5, 10, 20, 50 cm depth)
copper_active_area_surfaces	Outer surfaces of the active copper part (Domain 11) exposed to ambient temperature (soil thermal heat sources at 50 cm depth)

**Table 6:** Load parameters of the system modes.

$V_{\mathrm{LDO}}$	
MCU sleep mode (case 1)	$P_{\mathrm{SLEEP}}=3.63\;\mu W$
MCU sleep mode (case 2)	$P_{\mathrm{SLEEP}}=33.7\;\mu W$
MCU run	$P_{\mathrm{MCU}}=1.18\;\mu W$
MCU write to memory	$E_{\mathrm{MEM}}=11\;\mu J$
$V_{\mathrm{OUT}}$	
Measurement	$E_{\mathrm{MEA}}=99\;\mathrm{mJ}$
LoRaWAN transmission	$E_{\mathrm{TX}}=68.5\;\mathrm{mJ}$
Total time for measurement and transmission	$t_{\mathrm{CYCLE}}=12.3\;s$

**Table 7:** Statistical parameters of daily harvested energy, daily average electrical power and calculated output current for the specified voltage.

	|ΔT|	*E* (J/day)	*P* (μW)	*I* (μA) for $V_{\mathrm{OUT}}=2.35$ V
mean	1.40	11.15	129.04	54.91
25th	0.64	0.00	0.00	0.00
50th	1.23	4.82	55.78	23.74
75th	2.05	17.12	198.15	84.32
maximum	4.58	65.00	752.27	320.11

**Table 8:** Results of the simulated configurations. A missed cycle is a period during which transmission was required but energy was insufficient. The ratio represents the percentage of successful cycles and total periods. $E_{U}$ is unused energy. TTD represents the time to discharge, and overheads represent consumption during sleep.

Case	$I_{\mathrm{SLEEP}}$	Period	Complete	Missed	Ratio	Max. Delay	$E_{U}$	TTD	Overheads
	(μA)	(min)	(-)	(-)	(%)	(days)	(%)	(days)	(%)
1	1.65	10	18,751	33,810	35.7	10.0	17.3	0.42	1.2
2	1.65	60	6690	2070	76.4	7.5	66.7	2.37	6.7
3	1.65	240	2155	35	98.4	2.0	86.4	7.88	22.4
4	15.3	10	17,848	34,713	34.0	10.0	14.4	0.38	9.9
5	15.3	60	5588	3172	63.8	8.7	55.8	1.52	40.0
6	15.3	240	1723	467	78.7	7.3	69.5	2.76	72.8
